# Toward New Therapeutics for Skin and Soft Tissue Infections: Propargyl-Linked Antifolates Are Potent Inhibitors of MRSA and *Streptococcus pyogenes*

**DOI:** 10.1371/journal.pone.0029434

**Published:** 2012-02-07

**Authors:** Kishore Viswanathan, Kathleen M. Frey, Eric W. Scocchera, Brooke D. Martin, P. Whitney Swain III, Jeremy B. Alverson, Nigel D. Priestley, Amy C. Anderson, Dennis L. Wright

**Affiliations:** 1 Department of Pharmaceutical Sciences, University of Connecticut, Storrs, Connecticut, United States of America; 2 Promiliad Biopharma Inc., Alberton, Montana, United States of America; The Scripps Research Institute, United States of America

## Abstract

Hospital- and community-acquired, complicated skin and soft tissue infections, often attributed to *Staphylococcus aureus* and *Streptococcus pyogenes*, present a significant health burden that is associated with increased health care costs and mortality. As these two species are difficult to discern on diagnosis and are associated with differential profiles of drug resistance, the development of an efficacious antibacterial agent that targets both organisms is a high priority. Herein we describe a structure-based drug development effort that has produced highly potent inhibitors of dihydrofolate reductase from both species. Optimized propargyl-linked antifolates containing a key pyridyl substituent display antibacterial activity against both methicillin-resistant *S. aureus* and *S. pyogenes* at MIC values below 0.1 µg/mL and minimal cytotoxicity against mammalian cells. Further evaluation against a panel of clinical isolates shows good efficacy against a range of important phenotypes such as hospital- and community-acquired strains as well as strains resistant to vancomycin.

## Introduction

*Staphylococcus aureus* is a major cause of hospital-acquired infections, most frequently associated with the bloodstream, skin and soft tissue, ventilator-assisted pneumonia and catheters. The increasing frequency of infections caused by methicillin-resistant *S. aureus* (MRSA) is of particular concern, especially in the United States where the prevalence is more than 55% in the intensive care unit [1] and the incidence causes longer hospital stays, higher costs and higher risk of death [2]. Community-acquired MRSA (CA-MRSA), genotypically distinct from HA-MRSA, has also now become an established threat among patients without traditional risk factors [3], [4].

While vancomycin is the preferred treatment for MRSA infection in hospitals, vancomycin-intermediate *S. aureus* isolates (VISA) and vancomycin-resistant *S. aureus* (VRSA) strains have been reported in the US [5], [6] since 2002. Many strains of *S. aureus*, including new strains of CA-MRSA, show sensitivity to trimethoprim-sulfamethoxazole (TMP-SMZ). Unfortunately, resistance to TMP-SMZ among staphylococci has been observed in Australia and the United States since the early 1980s. Surveys from a collection of strains show that 28% of MRSA isolates are TMP-resistant and 35% are SMZ-resistant [7]. The importance of advancing improved antifolates to target MRSA is widely recognized and prompted the development of iclaprim, a highly potent antibiotic against Gram-positive bacteria [8], [9], [10]. Iclaprim reached Phase III clinical trials but was denied approval in 2009 pending additional clinical data to demonstrate efficacy and safety [11].

In addition to *S. aureus*, the Gram-positive bacteria *Streptococcus pyogenes* is a major cause of complicated skin and skin structure infections (SSTI). Reliably distinguishing between infections caused by these two agents is difficult because of overlaps in clinical presentation [12], [13]. Unfortunately, the spectrum of agents that may be effective against both *S. aureus* and *S. pyogenes* is limited by resistance. While *S. pyogenes* is frequently treated with beta-lactams, *S. aureus* shows widespread resistance to this class [5], [14]. Likewise, both strains can be resistant to macrolides [6], [15], [16]. Therapeutics with activity against MRSA and *S. pyogenes* would be ideal agents for treating SSTI.

Dihydrofolate reductase (DHFR) is a critical enzyme in the recycling of folate cofactors that are essential for the synthesis of deoxythymidine monophosphate and several amino acids. Since inhibition of DHFR depletes the pool of available thymidine, it has proven to be an excellent drug target for rapidly proliferating bacteria, protozoa and cancer cells. Despite the validation of DHFR as a drug target, TMP remains the only approved antibacterial inhibitor, targeting important pathogens such as MRSA for which it displays bactericidal activity [8], [17], [18]. Many pathogens have DHFR enzymes that are naturally resistant to TMP and several others are affected by point mutations that lead to TMP resistance.

Using high resolution structural information, we have developed a new class of antifolates characterized by a unique propargylic linker that shows activity against an expanded set of enzymes from important pathogens. Compounds in this series were shown to exhibit potent inhibition of wild-type MRSA DHFR as well as a critical resistance mutant, F98Y, known to introduce TMP insensitivity [19]. We anticipated that further evolution of this series could lead to compounds that are highly potent against wild-type MRSA and *S. pyogenes* DHFR. Herein, we present a new generation of propargyl-linked inhibitors with a critical pyridyl substitution that possess significant antibacterial activity (MIC values of 0.01 µg/mL and 0.09 µg/mL against MRSA and *S. pyogenes*, respectively). Crystal structures of representative members reveal productive contacts with active site residues and evaluation against a panel of clinical isolates of MRSA shows a spectrum of activity against clinically relevant phenotypes.

## Results and Discussion

**Figure 1:**
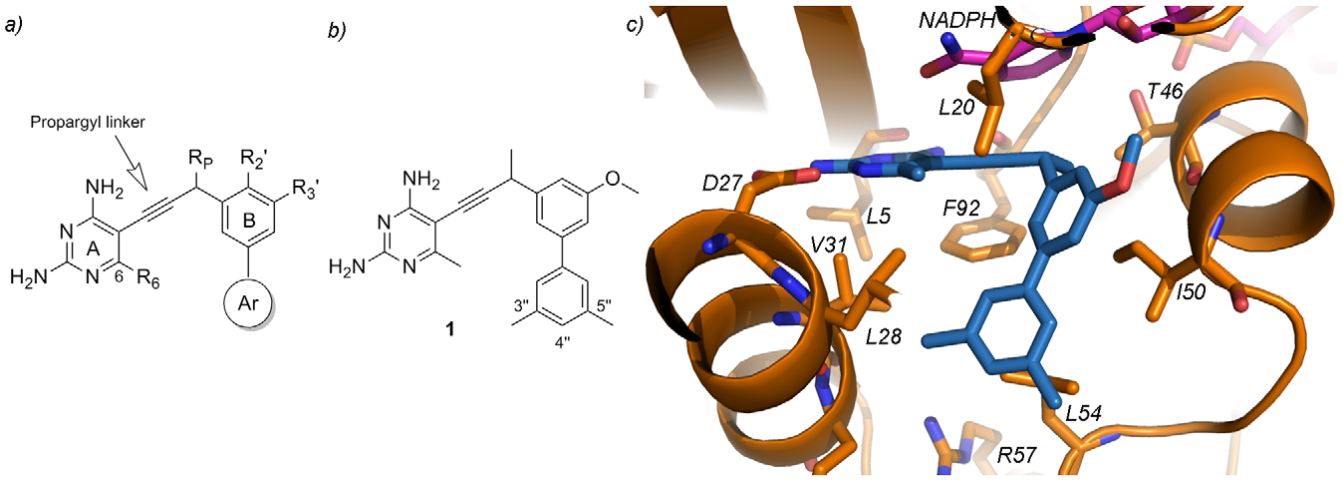
Propargyl-linked antifolates potently bind *S. aureus* DHFR. a) Depiction of a general scaffold for the propargyl-linked antifolates with the pyrimidine ring (A), phenyl ring (B) and aryl ring (Ar) shown along with possible positions for substitutions (R_6_, R_P_, R_2′_ and R_3′_) b) Illustration of compound **1**, a biphenyl propargyl-linked antifolate, with labeled atom positions b) Active site depiction from the structure of the SaDHFR:NADPH:**1** ternary complex showing active site residues (orange), NADPH (magenta) and compound **1** (blue).

Previous work had shown that a meta-biphenyl series of propargyl-linked antifolates (Figure 1a) were very promising inhibitors of the wild-type and F98Y mutant of *S. aureus* DHFR [19]. Specifically, compound **1** (Figure 1b) was the most potent in the series with an IC_50_ value of 42 nM against wild-type SaDHFR (Table 1) and moderate level of antibacterial activity (MIC value of 5.8 µg/mL, see Table 2). Further evaluation of this compound against the *S. pyogenes* DHFR enzyme reveals an IC_50_ value of 190 nM, suggesting that a compound based on the propargyl design could potentially target both enzymes. Importantly, compound **1** displays very good antibacterial activity against *S. pyogenes* with a MIC value of 0.1 µg/mL, demonstrating that *S. pyogenes* is also sensitive to these antifolate inhibitors. Furthermore, mammalian cytotoxicity against MCF-10 cells shows an eight-fold and 484-fold selectivity for MRSA and *S. pyogenes*, respectively. In order to advance this class of compounds, we sought to improve the antibacterial activity against MRSA without compromising the activity against *S. pyogenes* while ideally reducing cytotoxicity.

**Table 1:** Propargyl-linked DHFR inhibitors^a^ inhibit the *S. aureus* and *S. pyogenes* DHFR enzymes.

Number	Ar	R_6_	R_P_	R_2′_	R_3′_	IC_50_ (nM)			Selectivity	
						Sa^b^	Sp^b^	Human^b^	(h/Sa)^c^	(h/Sp)^c^
**1**	3,5-dimethyl phenyl	Me	Me	H	OMe	42±2	190±15	750±6	18	4
**2**	4-methyl phenyl	Me	Me	H	OMe	410±36	350±44	1400±15	3	4
**7**	phenyl	Et	H	OMe	H	2.4±0.2	5.9±0.2	300±10	125	51
**8**	phenyl	Et	H	H	H	28±1.7	26±1.7	290±36	10	11
**9**	phenyl	Et	H	H	OMe	59±2.3	52±1.7	140±1.0	2.4	3
**10**	phenyl	Me	Me	OMe	H	75±4	23±1.5	97±10	1.3	4
**11**	phenyl	Et	Me	OMe	H	67±5.5	26±2.5	100±10	1.5	4
**24**	4-pyridyl	Me	Me	H	OMe	26±2	160±9	1500±8	58	9.4
**25**	4-pyridyl	Et	Me	H	OMe	19±1	180±19	1300±11	68	7.2
**26**	4-pyridyl	Et	H	OMe	H	21±1.0	19±1.3	330±12	16	17
**27**	4-pyridyl	Et	H	H	OMe	12±2.4	28±4.0	61±5.7	5	2
**28**	4-pyridyl	Et	H	H	H	20±0.5	30±1.7	520±23	26	17
**31**	morphilino	Me	Me	H	OMe	29±2	26±4	400±40	14	15
**36**	3-pyridyl	Et	H	H	H	33±0.5	47±4.6	290±15	9	6
**37**	4-pyrimidinyl	Et	H	H	H	35±1.1	23±1.2	160±13	5	7
TMP						23	13000	198000	8600	15

a The generalized scaffold for the propargyl-linked inhibitors is shown in Figure 1a.

b IC_50_ values against the DHFR enzymes are reported in nM and represent the average of at least three measurements.

c Selectivity is calculated as IC_50_ (human)/IC_50_ (pathogen).

**Table 2:** Evaluation of antibacterial and cytotoxicity activity of propargyl-linked antifolates.

Cmpd #	MICMRSA^a^	MICMRSA^b^+FCS	MIC*S.pyogenes*	MIC*S. pyogenes*+FCS^c^	IC_50_MCF-10(µM)	IC_50_ HepG2(µM)	Selectivity^d^ MRSA	Selectivity*S. pyogenes*
**1**	5.76 (16)	ND	0.097 (0.25)	ND	47	ND	3	188
**2**	0.71 (2)	ND	0.024 (0.064)	ND	55	ND	28	859
**7**	0.18 (1)	ND	0.006 (0.016)	ND	67	ND	67	4,187
**8**	0.66 (2)	5.2 (16)	0.66 (2)	2.6 (8)	32	ND	16	16
**9**	0.18 (0.5)	1.4 (4)	0.18 (0.5)	0.72 (2)	199	ND	398	398
**10**	0.72 (2)	11.4 (32)	0.18 (0.5)	5.7 (16)	38	94	19	76
**11**	0.74 (2)	11.9 (32)	0.74 (2)	6 (16)	54	77	27	27
**24**	0.04 (0.125)	0.09 (0.25)	0.04 (0.125)	0.09 (0.25)	220	233	1,760	1,760
**25**	0.09 (0.5)	ND	0.012 (0.032)	ND	85	171	170	2,656
**26**	0.02 (0.064)	0.09 (0.25)	0.04 (0.125)	0.09 (0.25)	217	199	3,391	1,736
**27**	0.04 (0.125)	0.09 (0.25)	0.04 (0.125)	0.09 (0.25)	409	465	3,272	3,272
**28**	0.08 (0.25)	0.16 (0.5)	0.04 (0.125)	0.09 (0.25)	475	>500	1,900	3,800
**31**	1.5 (4)	2.94 (8)	2.94 (8)	2.94 (8)	462	494	116	58
**36**	0.041 (0.125)	ND	0.08 (0.25)	ND	>500	>500	>4,000	>2,000
**37**	0.16 (0.5)	0.16 (0.5)	0.16 (0.5)	0.16 (0.5)	>500	ND	>1,000	>1,000
**TMP**	0.14 (0.5)	0.14 (0.5)	0.58 (2)	0.58 (2)	ND	ND	ND	ND

a MIC values for MRSA and *S. pyogenes* are reported in µg/mL (µM).

b MIC values for MRSA in the presence of 10% fetal calf serum (FCS) in µg/mL (µM).

c MIC values for *S. pyogenes* in the presence of 10% FCS in µg/mL (µM).

d Selectivity values are calculated as IC_50_ (MCF10)/MIC (pathogen), both values in µM. ND: not determined.

Two strategies to improve the activity against MRSA emerge. One strategy focuses on improving both potency and selectivity of enzyme inhibition while a complementary strategy focuses on striking a better balance between solubility and permeability for these hydrophobic compounds. Enacting either of these strategies is greatly facilitated by obtaining structural information for the complex with the lead compound **1**, presented here (Figure 1c), and related congeners [19], [20], [21]. Determination of a co-crystal structure of SaDHFR:NADPH:**1** (PDB ID: 3F0S; statistics are listed in Supplementary Information) reveals a number of regions for potential modification. Specifically, hydrophobic contacts between the C6 substituent on the pyrimidine ring and Val 31 and Leu 28 as well as potential interactions between B-ring substituents and Ser 49 or the ribose of NADPH can be optimized. Although the propargylic methyl group on compound **1** appears to form van der Waals interactions with Phe 92 and Thr 46, it may also contribute to steric crowding that can be relieved by elimination of the methyl group, which would also decrease the hydrophobicity of the compounds. In addition to these pockets deep within the active site, it seemed attractive to optimize interactions between the distal C-ring and the opening of the active site that is exposed to solvent in order to improve both affinity and solubility. In previous optimization, it was shown that incorporation of the aromatic C-ring greatly enhanced potency by providing van der Waals contacts with Leu 54, Ile 50, Leu 28 and Val 31. However, from analysis of the structures, it is clear that only a portion of the phenyl ring makes productive hydrophobic contacts and that the C3″-C4″-C5″ region projects toward the solvent interface and Arg 57, thus introducing destabilizing interactions. The effect of these destabilizing interactions are more pronounced with incorporation of a C4″ methyl group (compound **2** in Table 1; IC_50_ = 410 nM). Thus, the design of more efficacious compounds incorporates aspects to increase affinity and solubility.

**Figure 2:**
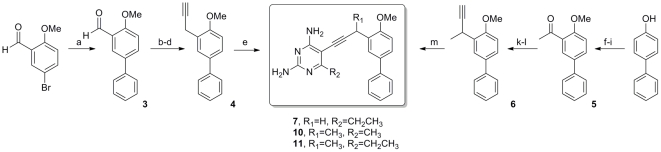
The synthesis of biphenyl analogs delivers compounds 7, 10 and 11. (a) PhB(OH)_2_, Pd(PPh_3_)_2_Cl_2_, Cs_2_CO_3_, dioxane, 80°C, 91%; (b) Ph_3_P = CHOMe, THF; (c) Hg(OAc)_2_, KI, THF/H_2_O; (d) dimethyl(1-diazo-2 oxopropyl)phosphonate, K_2_CO_3_, MeOH; 72% over 3 steps (e) 6-ethyl, 5-iodo-2,4-diaminopyrimidine, Pd(PPh_3_)_2_Cl_2_, CuI, Et_3_N, DMF, 81%; (f) Br_2_, CCl_4_, 65%; (g) KOH, MeI, DMSO, 90%; (h) n-butyllithium, acetaldehyde, 53%; (i) MnO_2_, 94%; (j) Ph_3_P = CHOMe, THF; (k) Hg(OAc)_2_, KI, THF/H2O; (l) dimethyl(1-diazo-2 oxopropyl)phosphonate, K_2_CO_3_, MeOH; 45% over 3 steps; (m) 6-alkyl, 5-iodo-2,4-diaminopyrimidine, Pd(PPh_3_)_2_Cl_2_, CuI, Et_3_N, DMF, 72%.

We have developed a modular synthetic approach to these propargyl-linked antifolates that relies on two versatile palladium-catalyzed cross-coupling reactions to efficiently assemble the inhibitors from three variable fragments [22], [23]. Characterization and purity data for all new inhibitors is included in Supporting Information S1. Three new biphenyl derivatives were prepared along analogous lines to give the final inhibitors in very good yield (Figure 2). 2-methoxy-5-bromobenzaldehyde was coupled to phenyl boronic acid and the resulting biphenyl aldehyde, **3**, was homologated to the terminal acetylene **4** through a three-step sequence that involved Wittig homologation to the phenylacetaldehyde followed by Ohira-Bestmann condensation to introduce the alkyne. A Sonogashira coupling to 6-ethyl, 5-iodo, 2,4-diaminopyrimidine produced the final inhibitor **7** in good yield. Biphenyl inhibitors **10** and **11** were achieved in a similar fashion from 4-hydroxybiphenyl. Regioselective bromination followed by methylation and acetylation gave **5**. The methyl ketone was homologated as previously described and the resultant alkyne **6** was coupled to the appropriate iodo-pyrimidine to yield inhibitors **10** and **11**.

**Figure 3:**
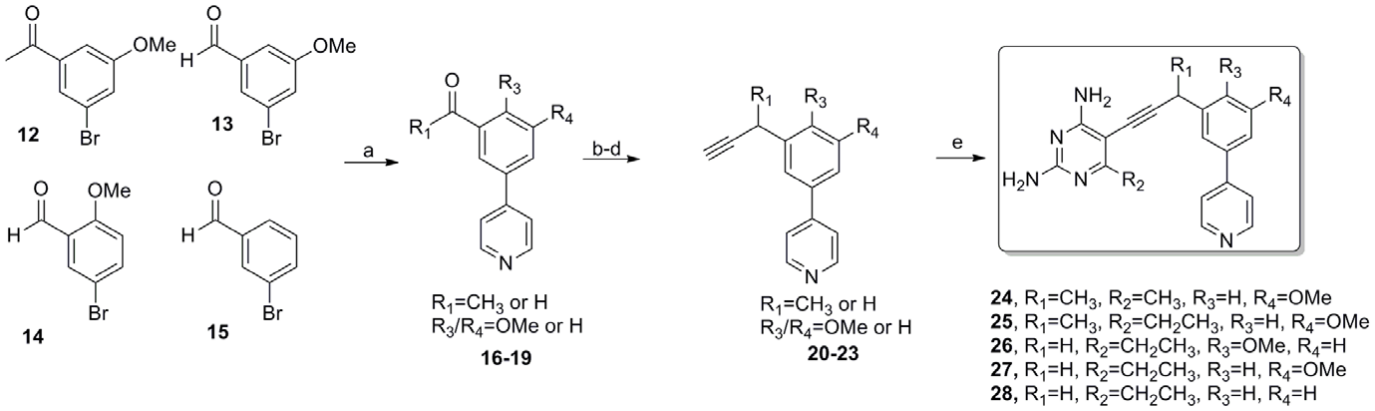
The synthesis of compounds with a pyridyl ring delivers compounds 24–28. (a) 4-pyridylboronic acid, Pd(PPh_3_)_2_Cl_2_, Cs_2_CO_3_, dioxane, 80°C, 82–89%; (b) Ph_3_P = CHOMe, THF; (c) Hg(OAc)_2_, KI, THF/H2O; (d) dimethyl(1-diazo-2 oxopropyl)phosphonate, K_2_CO_3_, MeOH; 61–70% over 3 steps (e) 6-alkyl, 5-iodo-2,4-diaminopyrimidine, Pd(PPh_3_)_2_Cl_2_, CuI, Et_3_N, DMF, 70–85%.

The introduction of a basic pyridine moiety into the biphenyl lead series was easily accomplished by modification of the previously described route (Figure 3). Suitably substituted bromo-benzaldehyde and bromo-acetophenone derivatives **12–15** could be cross-coupled in high yield with 4-pyridyl boronic acid to deliver biaryls **16**–**19** that were taken on through the three-step homologation to give the corresponding alkynes **20–23**. The presence of the basic pyridine group was well tolerated in the final palladium catalyzed cross-coupling to produce the pyridyl inhibitor series **24–28** for evaluation.

**Figure 4:**
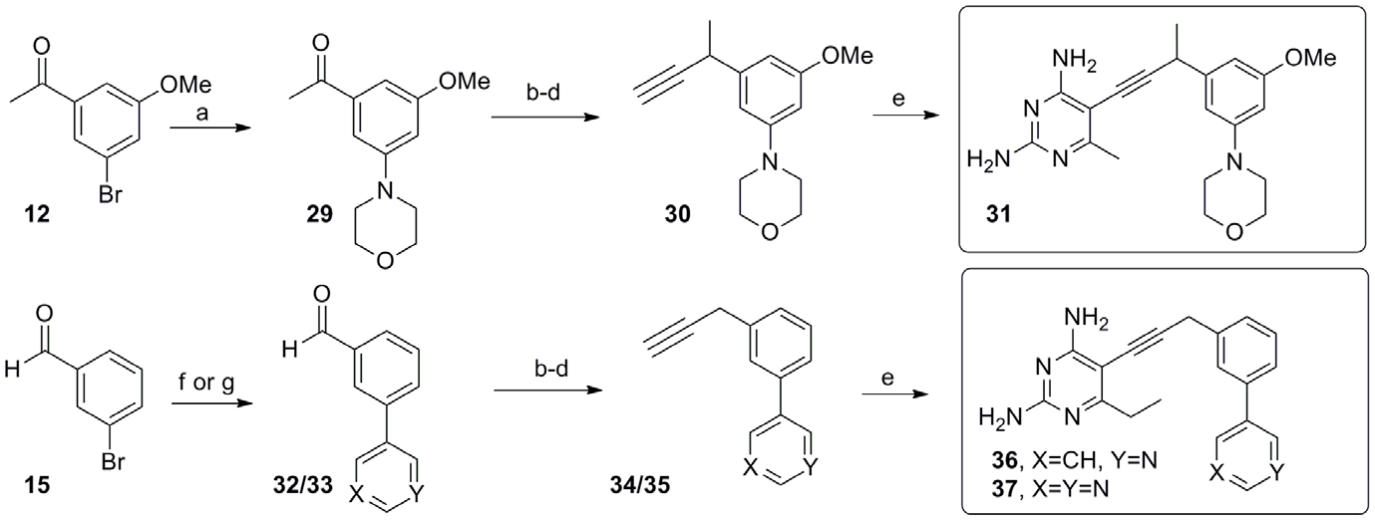
The synthesis of derivatives with alternative heterocycles delivers compounds 31, 36 and 37. (a) Pd(OAc)_2_, morpholine, Cs_2_CO_3_, (2-Biphenyl)di-tert-butyl phosphine, benzene, 80°C,92%; (b)Ph_3_P = CHOMe, THF; (c) Hg(OAc)_2_, KI, THF/H2O; (d) dimethyl(1-diazo-2-oxopropyl)phosphonate, K_2_CO_3_, MeOH, 57–69% over 3 steps; (e) 6-alkyl, 5-iodo-2,4-diaminopyrimidine, Pd(PPh_3_)_2_Cl_2_, CuI, Et_3_N, DMF, 68–82%, (f) 3-pyridylboronic acid, Pd(PPh_3_)_2_Cl_2_, Cs_2_CO_3_, dioxane, 80°C, 86%; (g) 4-pyrimidinylboronic acid, Pd(PPh_3_)_2_Cl_2_, Cs_2_CO_3_, dioxane, 80°C, 76%.

A final series of inhibitors with alternative heterocyclic functionality were accessed by further modification of this flexible, convergent route developed for this class of antifolates (Figure 4). Utilizing an amine in the coupling allowed for the introduction of a morpholine ring **29** while different boronate coupling partners permitted introduction of an isomeric 3-pyridyl or pyrimidinyl substituent as in **32**/**33**. Homologation to the terminal alkyne and cross-coupling to the iodopyrimidine building blocks delivered the final series of inhibitors **31**, **36** and **37**.

As solubility is an important consideration in drug development, a preferred lead series would exhibit solubility values greater than 20 µg/mL [24]. The heterocyclic series of derivatives (**24–28, 31, 36, 37**) were predicted to show improved solubility relative to the biphenyl series. In fact, solubility values were experimentally determined for a representative compound, **25**, and found to be in an acceptable range (40 µg/mL).

A series of biphenyl derivatives, **7–11**, probe the deletion of both propargylic and C-ring methyl groups, extension of the C6 alkyl group and the role of the B-ring alkoxy substituent. As predicted from the previous structural analysis, removal of the hydrophobic branching substituents both at the propargyl position and the distal aryl ring is well tolerated. Likewise, extension of the C6 alkyl chain had little impact on enzyme inhibition. However, the contribution of the B-ring substituent was more pronounced (compare compounds **7** and **9**), with a 2′-methoxy proving to be a more optimal placement, leading to a highly potent (2.4 nM and 5.9 nM) dual inhibitor of both *S. aureus* and *S. pyogenes* DHFR.

Interestingly, the structure-activity relationships observed in the biphenyl series did not directly translate to those analogs containing heterocyclic systems at ring C. Whereas the propargyl methyl and B-ring methoxy groups significantly impacted the activity of the biphenyl series, these substituents showed little effect in *S. aureus* DHFR across the heterocyclic series (compounds **24–28, 31, 36, 37**). In *S. pyogenes*, compounds without a propargyl methyl group exhibited 9-fold better enzyme inhibition (compare compounds **25** and **27**). In order to understand the divergence between activity in the biphenyl and heterocyclic series, crystal structures of *S. aureus* DHFR bound to compounds **7** or **25** were determined.

### Crystal structures of SaDHFR bound to NADPH and compounds 7 and 25

**Figure 5:**
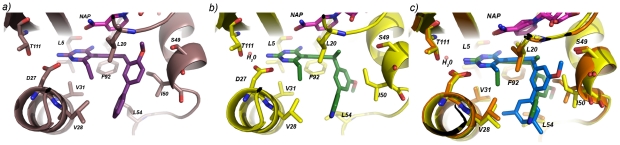
Depictions of *S. aureus* DHFR bound to ligands reveal interactions with active site residues. *S. aureus* DHFR is shown bound to NADPH (magenta) and a) compound **7** (purple), b) compound **25** (green) and c) a superposition of compounds **1** (blue) and **25** (green), as determined from crystal structures.

The structures of *S. aureus* DHFR bound to NADPH and **7** (Figure 5a; PDB ID: 3SH2; statistics in Table S1) or **25** (Figure 5b; PDB ID: 3SGY) were compared to the structure of *S. aureus* DHFR bound to compound **1**. Despite similarities between the three structures (the RMSD of Cα atoms is approximately 0.24 Å), there are several differences in ligand and residue conformations that explain the structure-activity relationships observed in the enzyme activity data (electron density depictions are shown in Figure S1). Comparison of the structures of *S. aureus* DHFR:NADPH:**7** and *S. aureus* DHFR:NADPH:**1** shows minimal changes in the orientation of the three aryl rings present in both inhibitors. However, a major difference that accounts for the 18-fold improvement in potency for compound **7** against *S. aureus* DHFR is the presence of a hydrogen bond (3.5 Å) between the 2′-methoxy substituent in compound **7** and the hydroxyl group of Ser 49, an interaction that is precluded with the 3′-methoxy in compound **1**.

Comparison of the structures of *S. aureus* DHFR:NADPH:**25** and *S. aureus* DHFR:NADPH:**1** (Figure 5c) reveals a dramatic reorientation of the hydrophobic regions of the pyridyl series relative to the parent biphenyl analogs. In fact, a different enantiomer of pyridine **25** preferentially crystallizes relative to the enantiomer observed with **1**. This new configuration forces a change in conformation for the nicotinamide ring of NADPH in order to maintain π-π stacking as well as a change in the conformation of Ile 50 that interacts with the proximal aryl ring. The stabilizing solvation of the pyridyl nitrogen at the opening of the active site drives the reorientation of compound **25** relative to compound **1**. One consequence of this reorientation is decreased contact between the B- and C-rings and the active site residues, which is reflected in the slight attenuation in enzyme inhibition.

### Antifolates with pyridyl substituents possess potent antibacterial activity

Evaluation of these new inhibitors in the propargyl-linked antifolate series against the targeted pathogens reveals that this series includes very potent antibacterial agents. Specifically, the pyridyl containing compounds possess MIC values below 0.1 µg/mL against both MRSA and *S. pyogenes*; several compounds possess superior activity when compared to TMP. Interestingly, evaluating the compounds in the presence of 10% serum highlighted the superiority of the pyridyl-substituted compounds relative to the initial biphenyl class. Whereas compounds containing biphenyl units (**8**–**11**) showed a 8-16-fold increase in the MIC value against MRSA in the presence of serum, the pyridyl derivatives showed minimal effect with serum and still maintain MIC values lower than 0.16 µg/mL (Table 2). The difference in sensitivity between the two classes with respect to the presence of serum likely reflects increased protein binding for the very hydrophobic biphenyl moiety. Addition of a basic nitrogen at this center decreases the hydrophobic character of this region and may reduce overall protein binding.

In order to validate that the antibacterial activity is a result of DHFR inhibition, we generated MRSA mutants resistant to a representative compound, **24**, by growing the organism in the presence of the inhibitor at ten times the MIC value. These conditions led to the isolation of resistant colonies that were further analyzed for changes to the DHFR gene sequence. Direct colony PCR of the DHFR gene revealed point mutations at the active site of DHFR, specifically Phe98Tyr, validating that DHFR is the primary target. Although this is the same point mutation known to confer resistance to TMP, the resistance frequency for compound **24** is very low (3.9×10^−10^). Additionally, compound **24** inhibits the growth of a MRSA strain containing the F98Y mutation with a MIC value of 2.5 µg/mL, which is considerably more potent than TMP that has a MIC value of 16 µg/mL against the F98Y strain [25].

In order to evaluate whether the compounds are bactericidal or bacteriostatic, we measured the minimum inhibitory and bactericidal concentrations (MBC) of compounds **24**, **25** and TMP in strain *Staphylococcus aureus* subsp. *aureus* ATCC 44300 (Table 3). From these results, the MIC values match the MBC values for compounds **24** and **25**, with both values equal to 0.078 µg/mL. Based on the MBC/MIC ratio of 1.0 for compounds **24** and **25**, which falls within the range of 1.0–4.0 cited for bactericidal antibiotics [26], there is some indication that these compounds may have bactericidal activity. Interestingly, for this strain, the MBC/MIC ratio for TMP is 2.0, indicating that it, too, may have bactericidal activity, agreeing with previous evidence [8], [17], [27].

**Table 3:** Evaluation of Minimum Bactericidal Concentrations.

Inhibitor	MIC value(µg/mL)	MBC value(µg/mL)	Ratio MBC/MIC
TMP	0.625	1.25	2.0
**24**	0.078	0.078	1.0
**25**	0.078	0.078	1.0

The heterocyclic series of antifolates are not only potent antibacterial agents but also possess minimal cytotoxicity when evaluated against two mammalian cell lines, MCF-10 and HepG2. Remarkably, the increased antibacterial activity of the heterocyclic series is not linked to mammalian cell toxicity, despite lower levels of enzyme selectivity (Table 1) in these derivatives. While the origin of the reduced cytotoxicity is unclear, one attractive possibility is that the compounds are sequestered in the lysosome. It has been shown that weakly basic compounds with a clogP value greater than 2 are subject to ion-trapping in the acidic environment of the lysosome [28], [29]. As the pyridyl containing analogs possess an additional basic nitrogen relative to the biphenyl series, they may have a greater propensity to accumulate in the lysosome, therefore minimizing inhibition of cytosolic mammalian DHFR. In fact, the compounds with a biphenyl at the C-ring position show a greater degree of cytotoxicity with IC_50_ values between 32–67 µM. Interestingly, lysosomal sequestration of antifolates has been previously reported as a mechanism of resistance in cancer chemotherapy [30], [31].

### Profiles against clinical isolates of MRSA

**Figure 6:**
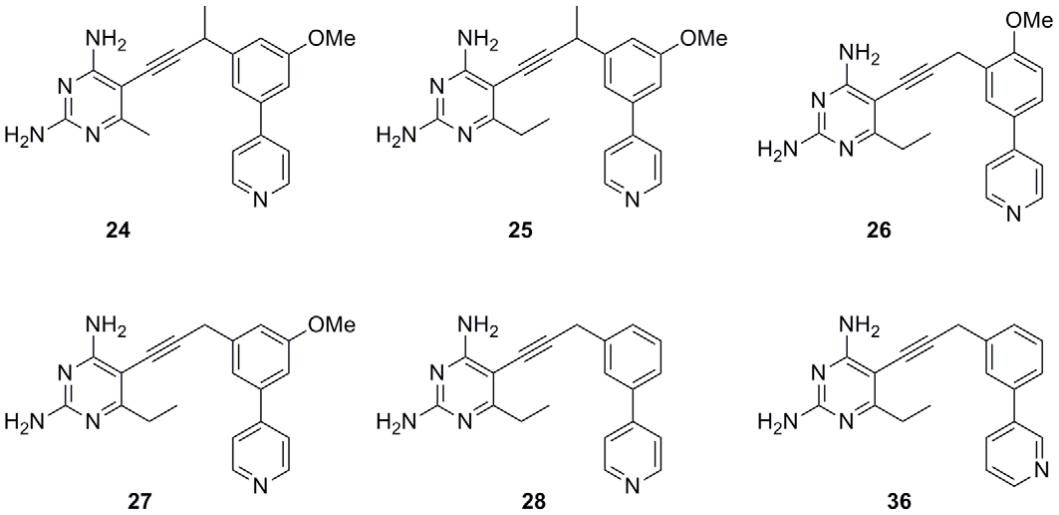
Active antifolates are evaluated against clinical isolates of MRSA.

As MRSA has globally evolved into a plethora of strains with varying phenotypes, it is important to evaluate the effects of promising compounds against a variety of clinically relevant isolates. Six propargyl-linked antifolates (**24–28**, **36**; Fig. 6) with the strongest antibacterial activity against *S. aureus* were selected for further evaluation against nine clinical isolates (Table 4). Of these isolates one strain is TMP-SMZ-resistant, two strains represent USA300 CA-MRSA, three strains represent USA100 HA-MRSA, one strain is HA-MRSA, and two strains are vancomycin-intermediate *S. aureus* (VISA; Mu3 and Mu50). The antibacterial effects of the propargyl-linked compounds were compared with the effects of vancomycin and TMP:SMZ controls. In all nine strains, the antibacterial activity of the propargyl-linked antifolates exceeded the activity of vancomycin. In eight strains, with the exception of one USA100 HA-MRSA strain, the antibacterial activity of the propargyl-linked antifolates also exceeded that of TMP:SMZ.

**Table 4:** Propargyl-linked antifolates inhibit the growth of a panel of MRSA strains (MIC values in µg/mL).

Compound (#)	TMP-SMZ^R^	USA300CA-MRSA	USA300CA-MRSA	USA100 HA-MRSA	USA100 HA-MRSA	USA100 HA-MRSA	HA-MRSA	hVISA^a^Mu3	VISAMu50
**24**	0.045	0.045	0.09	2.9	0.045	0.09	0.09	0.045	0.18
**25**	0.09	0.045	0.09	2.9	0.09	0.09	0.02	0.09	0.09
**26**	0.04	0.08	0.08	5.3	0.08	0.04	0.08	0.04	0.04
**27**	0.045	0.045	0.045	2.9	0.045	0.045	0.045	0.045	0.18
**28**	0.045	0.045	0.045	2.9	0.045	0.045	0.045	0.045	0.09
**36**	0.045	0.02	0.36	5.76	0.36	0.09	0.36	0.36	0.72
TMP∶SMZ(1∶20)^b^	1.5/30.5^c^	0.4/8	0.75/15	1.5/30.5	0.2/4	0.2/4	0.75/15	0.2/4	0.2/4
Vancomycin	2	1	1	0.5	1	1	1	3	8

a hVISA: heteroresistant vancomycin intermediate *S. aureus*.

b 1∶20 reflects the molar ratio of the two components.

c The two numbers reflect theindividual MIC values for each component of the mixture.

### Conclusions

Skin and soft tissue infections caused by *S. aureus* and *S. pyogenes* are a major health threat and as such, new antibiotics that treat both of these infections are critically needed. Using a structure-based approach, a series of propargyl-linked antifolates were designed, synthesized and shown to possess highly potent antibacterial activity against both MRSA and *S. pyogenes*. The incorporation of additional basic heterocycles not only improved the solubility of the compounds but also dramatically lowered mammalian cytotoxicity. Evaluation against a panel of clinical isolates reveals that the pyridyl-containing analogs are potent inhibitors of HA-MRSA, CA-MRSA and VISA strains at levels that exceed vancomycin or TMP-SMZ.

## Materials and Methods

### Enzyme Expression and Purification

*S. aureus* DHFR was cloned, expressed and purified as previously described [19]. Briefly, the DHFR gene was inserted into vector pET41 with a His_8_ tag for purification. The protein was expressed in *E. coli* BL21 (DE3) cells. The *S. aureus* DHFR protein was purified from all other proteins in the lysate, including *E. coli* DHFR, using nickel-affinity chromatography and desalted into 20 mM Tris (pH 8.0), 20% glycerol, 0.1 mM EDTA, 2 mM DTT using a PD-10 column (GE Healthcare). The protein was concentrated to ∼5 mg/mL. *S. pyogenes* DHFR was cloned using PCR from genomic DNA and inserted in vector pET-41. The protein was expressed and purified using the same procedures as detailed for *S. aureus* DHFR.

### Enzyme Inhibition Assays

Enzyme inhibition assays were performed by monitoring the rate of NADPH oxidation by the DHFR enzyme at an absorbance of 340 nm over five minutes. Assays were performed in the presence of saturating concentrations of NADPH (100 µM) and initiated with dihydrofolate (1 mM). All assays were completed at 25°C in a buffer containing 20 mM TES pH 7.0, 50 mM KCl, 10 mM 2-mercaptoethanol, 0.5 mM EDTA and 1 mg/mL BSA. Inhibition was measured at least three times with inhibitor concentrations near the IC_50_ value and the average IC_50_ value is reported with a standard deviation.

### Evaluation of Antibacterial Activity

Minimum inhibitory concentrations (MIC) were assessed for *S.aureus* 13709 and *Streptococcus pyogenes* using a broth microdilution approach based on CLSI standards and the use of the colorimetric reporter Alamar Blue. Stock solutions of standard antibiotics and test compounds were made at 50 mM in DMSO. Serial two-fold dilutions of the stocks were prepared in test wells with a maximum concentration of 500 mM (test concentrations therefore being 500, 250, 126, 64, 32, 16 µM etc.) MIC data are reported in µM and also converted into µg/mL for comparison to other literature data. For susceptibility testing, 10 µL of glycerol stock was suspended in a 10 mL shake flask culture of chemically defined Isosensitest broth (Oxoid) supplemented with 2% w/v glucose. A sample of the shake flask culture was diluted to 1×10^6^ cells/mL in media and added to 96-well test plates (100 µL per well) containing test compounds dispensed in DMSO (2 µL) using serial two-fold dilutions. After an incubation period (30°C) determined from the strain specific doubling time, a 0.03% w/v aqueous solution of resazurin (10 µL) was added and the plates were allowed to incubate; each well was then scored for dye reduction. These methods reproduce reported MIC values for standard antibiotics such as kanamycin, vancomycin and chloramphenicol. The MIC value was taken as the lowest concentration of test compound that inhibits growth such that less than 1% reduction of resazurin (λmax 570 nm) to resorufin (λmax 600 nm) was observed.

### Evaluation of Mammalian Cell Toxicity

Adherent cell lines were maintained in Eagle's Minimal Essential Media (Sigma-Aldrich, St. Louis, MO, USA) with 2 mM glutamine and Earle's Balanced Salt Solution (HyClone, Logan, UT, USA) adjusted to contain 1.5 g/L sodium bicarbonate, 0.1 mM non-essential amino acids, 1 mM sodium pyruvate and 10% fetal calf serum. Fetal calf serum used in these assays was lot matched throughout. All cultures were maintained under a humidified 5% CO_2_ atmosphere at 37°C, had media refreshed twice weekly and were subcultured by trypsinization and resuspension at a ratio of 1∶5 each week. Toxicity assays were conducted between passages 10–20. Target compound toxicity was measured by incubating the test compound with the cells for 4 h, washing the cells and finally treating the cells with Alamar Blue. After 12–24 h, the fluorescence of the reduced dye was measured. Fluorescence intensity as a function of test compound concentration was fit to the Fermi equation, using non-linear least squares regression analysis, to estimate IC_50_ values.

### Crystallization and Structure Determination

*S. aureus* DHFR was co-crystallized with **1**, **7**, and **25** using the hanging drop vaporization method. Protein (12 mg/mL) was incubated with ligand (1 mM) and NADPH (2 mM) for two hours on ice. An equal volume of the protein:ligand:NADPH complex was mixed with an optimized crystallization solution consisting of 15% PEG 10,000, 150 mM sodium acetate, 100 mM MES pH 6.5, and 5% butyrlactone (Sigma Aldrich). All crystal growth followed the same procedures and typically yielded crystals within 5–7 days. Crystals were incubated in a cryo-protectant buffer containing 15% glycerol then flash-cooled with liquid nitrogen. High resolution data sets were collected at Brookhaven NSLS on beamline ×25.

Data were indexed and scaled used HKL2000. Crystal structures for all complexes were solved using a model of *S. aureus* DHFR bound to folate. The diffraction data for the complex of SaDHFR:NADPH:**1** and the model share the same hexagonal space group (P6_1_22) and unit cell dimensions, therefore difference Fourier methods were used to solve the phase problem for these data. The crystals of SaDHFR:NADPH:**7** and SaDHFR:NADPH:**25** belong to space group P6_1_ and there are two molecules in the asymmetric unit. Therefore, these structures were determined by molecular replacement using Phaser [32] The programs COOT and Refmac5 were used to build and refine the structure until an acceptable R_factor_ and R_free_ were achieved (data and refinement statistics are noted in Table S1). Structure geometry was evaluated using Procheck and Ramachandran plots. Solvent was not included in the final models for the crystal structures of *S. aureus* DHFR bound to NADPH and compounds **1** and **7**.

### MBC determination for MRSA strain 43300

Minimal bactericidal concentrations (MBCs) were determined for *Staphylococcus aureus* subsp. *aureus* ATCC 44300 by performing MIC determinations using the microdilution method according to CLSI standards [33], then culturing the visibly clear wells (as confirmed by OD_600_) onto Isosensitest Agar (ISA; Oxoid) and growing for 24 hours at 37°C. MBC was defined as the lowest concentration of inhibitor preventing colony growth on agar plates [34].

### Selection of resistant MRSA colonies

For these experiments, MRSA strain ATCC 43300 was used as the progenitor strain. Resistant colonies were selected by plating 100 µL of saturated overnight culture (approximately 10^11^ CFU/mL) in triplicate on selective ISA containing 10× MIC concentration of compound **24** [25]. Selective plates were grown for 30 hours at 37°C. All colonies resistant to compound **24** were re-passaged on selective ISA. Direct colony PCR was used to isolate and amplify the *dfrA* gene from resistant strains using methods described in [34]. All PCR products were screened for point mutations in DHFR by high quality DNA sequencing (Genewiz). Primers used to detect the *dfrA* gene contained restriction enzyme cut sites for NdeI and XhoI in order to ligate PCR products into the pET41 vector for further validation with DNA sequencing with the T7 promoter sequencing primer. Mutation frequencies were calculated based on the sequencing information provided for each mutant colony.

## Supporting Information

Supporting Information S1 Characterization, spectral and purity data for compounds **7**, **10**, **11**, **24–28**, **31** and **36–37**.
(DOCX)

Figure S1 Electron density for the active site and ligands, calculated as Fo-Fc omit maps show a) compound **1** (1.3 σ), b) compound **7** (1.3 σ) and c) compound **25** (1.0 σ).
(TIF)

Table S1 Statistics for data collection and refinement of crystal structures.
(DOC)
