# Supplementary material for: Toward New Therapeutics for Skin and Soft Tissue Infections: Propargyl-Linked Antifolates Are Potent Inhibitors of MRSA and Streptococcus pyogenes
Source: PLoS One. 2012 Feb 7;7(2):e29434. doi: 10.1371/journal.pone.0029434 (PMC3274548; doi:10.1371/journal.pone.0029434)
Supplement: Supporting Information S1 — Characterization, spectral and purity data for compounds 7, 10, 11, 24–28, 31 and 36–37. (DOCX) [file pone.0029434.s001.docx]

Toward new therapeutics for skin and soft tissue infections: Propargyl-linked antifolates are potent inhibitors of MRSA and *Streptococcus pyogenes*

Kishore Viswanathan^1ǂ^, Kathleen M. Frey^1ǂ^, Eric W. Scocchera^1^, Brooke D. Martin^2^, P. Whitney Swain III^2^, Jeremy Alverson^2^, Nigel D. Priestley^2^, Amy C. Anderson^1^* and Dennis L. Wright^1^*

^1^Department of Pharmaceutical Sciences, University of Connecticut, 69 N. Eagleville Rd., Storrs, CT 06269 and ^2^ Promiliad Biopharma Inc., Alberton, MT 59820

**Contents:**

HPLC Method for Purity Determination **S2**

Synthetic Scheme, ^1^H NMR , ^13^C NMR, HPLC data for compounds **7, 10-19 S3-S8**

^1^H NMR and ^13^C NMR spectra for compounds **7, 10-19 S9-S29**

**HPLC Method :**

Purity analyses were accomplished with reversed-phase high performance liquid chromatography (RP-HPLC) performed on a Shimadzu Prominence 20 instrument fitted with a Luna 5μ C18(2) 100 Ǻ column (5 μM, 4.6 mm x 250 mm, Phenomenex) and using UV diode array detection at 254 nm. Two separate determinations (Methods A and B, described below) were made to determine compound purity. Flow rate was 1.5 mL/min for Method A and 1.0 mL/min for Method B.

Compounds were diluted in the HPLC grade mobile phase and filtered prior to analysis. Sample

concentrations were 50 μg/ml and injection volume was 10 μL.

Method A: isocratic- 40% (v/v) MeCN/ 60% (v/v) 50 mM Potassium Phosphate,

monobasic, pH=7.0

Method B: isocratic- 70% (v/v) MeOH/ 30% (v/v) 50 mM Potassium Phosphate,

monobasic, pH=7.0

.

1. Pd(PPh_3_)_2_Cl_2_, Cs_2_CO_3_, dioxane, 80°C, 91%; (b) Ph_3_P=CHOMe, THF; (c) Hg(OAc)_2_, KI, THF/H_2_O; (d) dimethyl(1-diazo-2 oxopropyl)phosphonate, K_2_CO_3_, MeOH; 72% over 3 steps (e) Pd(PPh_3_)_2_Cl_2_, CuI, Et_3_N, DMF, 81%.

**6-Ethyl-5-[3-(4-methoxy-biphenyl-3-yl)-prop-1-ynyl]-pyrimidine-2,4-diamine (7).** ^1^H NMR (500 MHz, CDCl_3_) δ 7.75 (d, *J* = 2.25 Hz, 1H), 7.56 (dd, *J* = 1.10, 8.17 Hz, 2H), 7.49 (dd, *J* = 2.33, 8.40 Hz, 1H), 7.41 (t, *J* = 7.72 Hz, 2H), 7.33 – 7.27 (m, 1H), 6.95 (d, *J* = 8.44 Hz, 1H), 5.25 (s, 2H), 4.92 (s, 2H), 3.90 (d, *J* = 2.71 Hz, 5H), 2.73 (q, *J* = 7.60 Hz, 2H), 1.23 (t, *J* = 7.59 Hz, 4H); ^13^C NMR (126 MHz, CDCl_3_) δ 173.04, 164.48, 160.51, 156.40, 140.74, 133.74, 128.75, 127.72, 126.76, 126.71, 126.50, 125.55, 110.45, 96.42, 75.35, 55.56, 29.57, 21.02, 12.58; HRMS (ESI, M^+^) *m/z* 359.1817 (calculated for C_22_H_23_N_4_O, 359.1872); HPLC (a) t_R_=12.4 min, 87.8%,

(a) Br_2_, CCl_4_, 65%; (b) KOH, MeI, DMSO, 90%; (c) n-butyllithium, acetaldehyde, 53%; (d) MnO_2_, 94%; (e) Ph_3_P=CHOMe, THF; (f) Hg(OAc)_2_, KI, THF/H_2_O; (g) dimethyl(1-diazo-2 oxopropyl)phosphonate, K_2_CO_3_, MeOH; 45% over 3 steps; (h) Pd(PPh_3_)_2_Cl_2_, CuI, Et_3_N, DMF, 72%.

**5-[3-(4-Methoxy-biphenyl-3-yl)-but-1-ynyl]-6-methyl-pyrimidine-2,4-diamine (10).** ^1^H NMR (500 MHz, CDCl_3_) δ 7.78 (s, 1H), 7.55 (d, *J* = 7.58 Hz, 2H), 7.47 (d, *J* = 8.40 Hz, 1H), 7.41 (t, *J* = 7.57 Hz, 2H), 7.30 (t, *J* = 7.35 Hz, 1H), 6.96 (d, *J* = 8.45 Hz, 1H), 5.35 (s, 2H), 5.15 (s, 2H), 4.45 (q, *J* = 6.98 Hz, 1H), 3.91 (s, 3H), 3.17 (q, *J* = 7.30 Hz, 2H), 2.42 (s, 3H), 1.57 (d, *J* = 7.01 Hz, 3H), 1.47 (t, *J* = 7.32 Hz, 3H); ^13^C NMR (126 MHz, CDCl_3_) δ 171.72, 167.67, 164.21, 160.05, 155.74, 140.80, 133.85, 131.70, 128.78, 126.78, 126.70, 126.51, 126.47, 110.94, 102.47, 91.82, 74.50, 55.62, 46.30, 27.09, 22.92; HRMS (ESI, M^+^) *m/z* 359.1901 (calculated for C_22_H_23_N_4_O, 359.1872); HPLC (a) t_R_=14.96 min, 98.9%, (b) t_R_ =42.37 min, 99.5%.

**6-Ethyl-5-[3-(4-methoxy-biphenyl-3-yl)-but-1-ynyl]-pyrimidine-2,4-diamine (11).** ^1^H NMR (500 MHz, CDCl_3_) δ 7.82 (d, *J* = 2.13 Hz, 1H), 7.56 (d, *J* = 7.42 Hz, 2H), 7.47 (dd, *J* = 2.20, 8.39 Hz, 1H), 7.41 (t, *J* = 7.62 Hz, 2H), 7.30 (t, *J* = 7.30 Hz, 1H), 6.96 (d, *J* = 8.44 Hz, 1H), 5.23 (s, 2H), 4.93 (s, 2H), 4.47 (q, *J* = 6.93 Hz, 1H), 3.91 (s, 3H), 2.72 (q, *J* = 7.57 Hz, 2H), 1.57 (d, *J* = 6.99 Hz, 3H), 1.23 (t, *J* = 7.58 Hz, 4H); ^13^C NMR (126 MHz, CDCl_3_) δ 173.28, 164.39, 160.69, 155.72, 151.36, 140.84, 133.88, 131.84, 128.76, 126.76, 126.73, 126.56, 126.41, 110.88, 102.04, 90.84, 74.65, 55.59, 29.69, 27.08, 23.01, 12.53; HRMS (ESI, M^+^) *m/z* 373.2024 (calculated for C_23_H_25_N_4_O, 373.2028); HPLC (a) t_R_=20.95 min, 98.8%, (b) t_R_ =56.81 min, 99.9%.

1. Pd(PPh_3_)_2_Cl_2_, Cs_2_CO_3_, dioxane, 80°C, 89%; (b) Ph_3_P=CHOMe, THF; (c) Hg(OAc)_2_, KI, THF/H_2_O; (d) dimethyl(1-diazo-2 oxopropyl)phosphonate, K_2_CO_3_, MeOH; 70% over 3 steps (e) Pd(PPh_3_)_2_Cl_2_, CuI, Et_3_N, DMF.

**5-[3-(3-Methoxy-5-pyridin-4yl-phenyl)-but-1-ynyl]-6-methyl-pyrimidine-2,4-diamine (12).** ^1^H NMR (500 MHz, CDCl_3_) δ 8.66(dd, *J* = 1.60, 4.49 Hz, 2H), 7.49 (dd, *J* = 1.64, 4.51 Hz, 2H), 7.27 (s, 1H), 7.09 – 7.06 (m, 1H), 7.06 – 7.03 (m, 1H), 5.07 (bs, 2H), 4.77 (bs, 2H), 4.10 (q, *J* = 7.11 Hz, 1H), 3.89 (s, 3H), 2.39 (s, 3H), 1.63 (d, *J* = 7.13 Hz, 3H); ^13^C NMR (125 MHz, CDCl_3_) δ 168.55, 164.09, 160.48, 160.40, 150.27, 148.14, 145.89, 139.98, 121.71, 118.01, 113.15, 111.06, 101.35, 91.26, 75.92, 55.46, 33.12, 24.71, 22.82; HRMS (ESI, M^+^) *m/z* 360.1832 (calculated for C_21_H_22_N_5_O, 360.1819); HPLC (a) t_R_=3.45 min, 98.6%, (b) t_R_ =10.15 min, 99.7%.

**5-[3-(3-Methoxy-5-pyridin-4yl-phenyl)-but-1-ynyl]-6-ethyl-pyrimidine-2,4-diamine (13).** ^1^H NMR (500 MHz, CDCl_3_) δ 8.66 (dd, *J* = 1.58, 4.49 Hz, 2H), 7.49 (dd, *J* = 1.65, 4.50 Hz, 2H), 7.28 (s, 1H), 7.07 (d, *J* = 1.80 Hz, 1H), 7.04 (d, *J* = 1.59 Hz, 1H), 5.09 (bs, 2H), 4.82 (bs, 2H), 4.09 (q, *J* = 7.05 Hz, 1H), 3.89 (s, 3H), 2.71 (q, *J* = 7.58 Hz, 2H), 1.63 (d, *J* = 7.09 Hz, 3H), 1.23 (t, *J* = 7.58 Hz, 3H); ^13^C NMR (125 MHz, CDCl_3_) δ 173.45, 164.24, 160.69, 160.46, 150.25, 148.13, 145.91, 139.97, 121.73, 117.99, 113.11, 111.04, 101.09, 90.36, 75.75, 55.45, 33.13, 29.66, 24.71, 12.54; HRMS (ESI, M^+^) *m/z* 374.1995 (calculated for C_22_H_24_N_5_O, 374.1975); (a) t_R_=4.25 min, 99%, (b) t_R_ =13.04 min, 99.4%.

1. Pd(PPh_3_)_2_Cl_2_, Cs_2_CO_3_, dioxane, 80°C, 82%; (b) Ph_3_P=CHOMe, THF; (c) Hg(OAc)_2_, KI, THF/H_2_O; (d) dimethyl(1-diazo-2 oxopropyl)phosphonate, K_2_CO_3_, MeOH; 61% over 3 steps (e) Pd(PPh_3_)_2_Cl_2_, CuI, Et_3_N, DMF, 74%.

**5-[3-(2-Methoxy-5-pyridin-4yl-phenyl)-prop-1-ynyl]-6-ethyl-pyrimidine-2,4-diamine (14).**^1^H NMR (500 MHz, CDCl_3_) δ 8.62 (s, 2H), 7.81 (d, *J* = 2.22 Hz, 1H), 7.57 (dd, *J* = 2.34, 8.47 Hz, 1H), 7.48 (d, *J* = 5.03 Hz, 2H), 6.99 (d, *J* = 8.50 Hz, 1H), 5.21 (bs, 2H), 4.94 (bs, 2H), 3.93 (s, *J* = 4.91 Hz, 3H), 3.91 (s, 2H), 2.73 (q, *J* = 7.60 Hz, 2H), 1.24 (t, *J* = 7.58 Hz, 3H); ^13^C NMR (126 MHz, CDCl_3_) δ 173.33, 164.48, 160.67, 157.78, 150.25, 147.78, 130.34, 127.48, 126.65, 126.15, 121.06, 110.61, 95.90, 93.58, 90.70, 55.63, 29.64, 21.02, 12.62; HRMS (ESI, M^+^) *m/z* 360.1842 (calculated for  C_21_H_22_N_5_O, 360.1824); HPLC (a) t_R_=8.02 min, 94.6%, (b) t_R_ =11.08 min, 94.1%.

1. Pd(PPh_3_)_2_Cl_2_, Cs_2_CO_3_, dioxane, 80°C, 84%; (b) Ph_3_P=CHOMe, THF; (c) Hg(OAc)_2_, KI, THF/H_2_O; (d) dimethyl(1-diazo-2 oxopropyl)phosphonate, K_2_CO_3_, MeOH; 65% over 3 steps (e) Pd(PPh_3_)_2_Cl_2_, CuI, Et_3_N, DMF, 77%.

**5-[3-(3-Methoxy-5-pyridin-4yl-phenyl)-prop-1-ynyl]-6-ethyl-pyrimidine-2,4-diamine (15).**^1^H NMR (500 MHz, DMSO) δ 8.56 (s, 2H), 7.61 (s, 2H), 7.30 (s, 1H), 7.11 (s, 1H), 7.00 (s, 1H), 6.38 (bs, 2H), 6.15 (s, 2H), 3.88 (s, 2H), 3.74 (s, 3H), 2.46 (q, *J* = 7.56 Hz, 2H), 1.00 (t, *J* = 7.57 Hz, 3H); ^13^C NMR (125 MHz, DMSO) δ 164.91, 160.53, 150.66, 147.39, 140.42, 139.17, 121.83, 119.15, 114.87, 110.90, 96.13, 88.60, 79.61, 76.48, 55.80, 28.99, 26.04, 12.92; HRMS (ESI, M^+^) *m/z* 360.1797 (calculated for  C_21_H_22_N_5_O, 360.1824); HPLC (a) t_R_=6.81 min, 96.6%, (b) t_R_ =9.29 min, 97.5%.

1. Pd(PPh_3_)_2_Cl_2_, Cs_2_CO_3_, dioxane, 80°C, 88%; (b) Ph_3_P=CHOMe, THF; (c) Hg(OAc)_2_, KI, THF/H_2_O; (d) dimethyl(1-diazo-2 oxopropyl)phosphonate, K_2_CO_3_, MeOH; 63% over 3 steps; (e) Pd(PPh_3_)_2_Cl_2_, CuI, Et_3_N, DMF, 70%.

**5-[3-(3-pyridin-4yl-phenyl)-prop-1-ynyl]-6-ethyl-pyrimidine-2,4-diamine (16).**^1^H NMR (500 MHz, DMSO) δ 8.64 (d, *J* = 5.61 Hz, 2H), 7.80 (d, *J* = 29.67 Hz, 1H), 7.69 (dd, *J* = 3.22, 7.93 Hz, 3H), 7.54 – 7.48 (m, 2H), 6.33 (bs, 2H), 6.15 (s, 2H), 4.01 (s, 2H), 2.55 (q, *J* = 7.54 Hz, 2H), 1.10 (t, *J* = 7.55 Hz, 3H); ^13^C NMR (125 MHz, DMSO) δ 172.14, 164.93, 161.70, 150.74, 147.44, 139.07, 137.82, 129.85, 129.20, 126.76, 125.47, 121.68, 95.94, 88.27, 76.94, 29.34, 26.02, 12.96; HRMS (ESI, M^+^) *m/z* 330.1723 (calculated for  C_20_H_20_N_5_, 330.1718); HPLC (a) t_R_=6.40 min, 96.8%, (b) t_R_ =8.50 min, 96.6%.

1. Pd(OAc)_2_, morpholine, Cs_2_CO_3_, (2-Biphenyl)di-tert-butyl phosphine, benzene, 80°C,92%; (b)Ph_3_P=CHOMe, THF; (c) Hg(OAc)_2_, KI, THF/H_2_O; (d) dimethyl(1-diazo-2-oxopropyl)phosphonate, K_2_CO_3_, MeOH, 69% over 3 steps; (e) Pd(PPh_3_)_2_Cl_2_, CuI, Et_3_N, DMF, 82%.

**5-[3-(3-Methoxy-5-morpholin-4yl-phenyl)-but-1-ynyl]-6-methyl-pyrimidine-2,4-diamine (17).** ^1^H NMR (500 MHz, CDCl_3_) δ 6.60 (s, 1H), 6.52 (s, 1H), 6.34 (t, *J* = 2.09 Hz, 1H), 5.08 (bs, 2H), 4.79 (bs, 2H), 3.96 (q, *J* = 7.05 Hz, 1H), 3.84 (m, 4H), 3.81 (s, 3H), 3.21 – 3.11 (m, 4H), 2.38 (s, 3H), 1.58 (d, *J* = 7.13 Hz, 3H); ^13^C NMR (125 MHz, CDCl_3_) δ 168.39, 164.13, 160.88, 160.33, 152.83, 145.56, 106.99, 103.68, 102.00, 100.39, 91.57, 75.44, 66.88, 55.27, 49.29, 33.48, 24.74, 22.84; HRMS (ESI, M^+^) *m/z* 368.2095 (calculated for C_20_H_26_N_5_O_2_, 368.2081); HPLC (a) t_R_=3.53 min, 96.3%, (b) t_R_ =8.36 min, 97.5%.

1. Pd(PPh_3_)_2_Cl_2_, Cs_2_CO_3_, dioxane, 80°C, 86%; (b) Ph_3_P=CHOMe, THF; (c) Hg(OAc)_2_, KI, THF/H_2_O; (d) dimethyl(1-diazo-2 oxopropyl)phosphonate, K_2_CO_3_, MeOH; 58% over 3 steps (e) Pd(PPh_3_)_2_Cl_2_, CuI, Et_3_N, DMF, 72%.

**5-[3-(3-pyridin-3yl-phenyl)-prop-1-ynyl]-6-ethyl-pyrimidine-2,4-diamine (18).**^1^H NMR (500 MHz, DMSO) δ 8.88 (d, *J* = 1.46 Hz, 1H), 8.58 (d, *J* = 3.71 Hz, 1H), 8.08 – 8.04 (m, 1H), 7.75 (s, 1H), 7.60 (d, *J* = 6.89 Hz, 1H), 7.53 – 7.44 (m, 3H), 6.38 (bs, 2H), 6.19 (s, 2H), 4.00 (s, 2H), 2.55 (q, *J* = 7.56 Hz, 2H), 1.09 (t, *J* = 7.56 Hz, 3H); ^13^C NMR (125 MHz, DMSO) δ 171.81, 164.92, 161.48, 149.04, 148.10, 138.92, 137.73, 136.01, 134.56, 129.77, 128.11, 126.87, 125.54, 124.37, 96.09, 88.39, 76.75, 29.24, 26.04, 12.95; HRMS (ESI, M^+^) *m/z* 330.1706 (calculated for  C_20_H_20_N_5_, 330.1718); HPLC (a) t_R_=6.53 min, 98.3%, (b) t_R_ =8.49 min, 95.6%.

1. Pd(PPh_3_)_2_Cl_2_, Cs_2_CO_3_, dioxane, 80°C, 76%; (b) Ph_3_P=CHOMe, THF; (c) Hg(OAc)_2_, KI, THF/H_2_O; (d) dimethyl(1-diazo-2 oxopropyl)phosphonate, K_2_CO_3_, MeOH; 57% over 3 steps (e) Pd(PPh_3_)_2_Cl_2_, CuI, Et_3_N, DMF, 68%.

**5-[3-(3-pyrimidin-5yl-phenyl)-prop-1-ynyl]-6-ethyl-pyrimidine-2,4-diamine (19).**^1^H NMR (500 MHz, CDCl_3_) δ 9.22 (s, 1H), 8.96 (s, 2H), 7.60 (s, 1H), 7.52 (d, *J* = 4.74 Hz, 2H), 7.49 (d, *J* = 5.03 Hz, 1H), 5.16 (bs, 2H), 4.94 (bs, 2H), 4.00 (s, 2H), 2.72 (q, *J* = 7.60 Hz, 2H), 1.24 (t, *J* = 7.59 Hz, 3H); ^13^C NMR (125 MHz, MeOD/CDCl_3_) δ 172.22, 164.53, 160.35, 156.64, 154.89, 138.52, 134.75, 134.06, 129.72, 128.72, 126.38, 125.38, 95.83, 90.09, 75.15, 29.09, 25.76, 12.30; HRMS (ESI, M^+^) *m/z* 331.1688 (calculated for  C_19_H_19_N_6_, 331.1671); HPLC (a) t_R_=4.18 min, 94.5%, (b) t_R_ =6.02 min, 95.9%.
