# Supplementary figures and images for: Toward New Therapeutics for Skin and Soft Tissue Infections: Propargyl-Linked Antifolates Are Potent Inhibitors of MRSA and Streptococcus pyogenes
Source: PLoS One. 2012 Feb 7;7(2):e29434. doi: 10.1371/journal.pone.0029434 (PMC3274548; doi:10.1371/journal.pone.0029434)

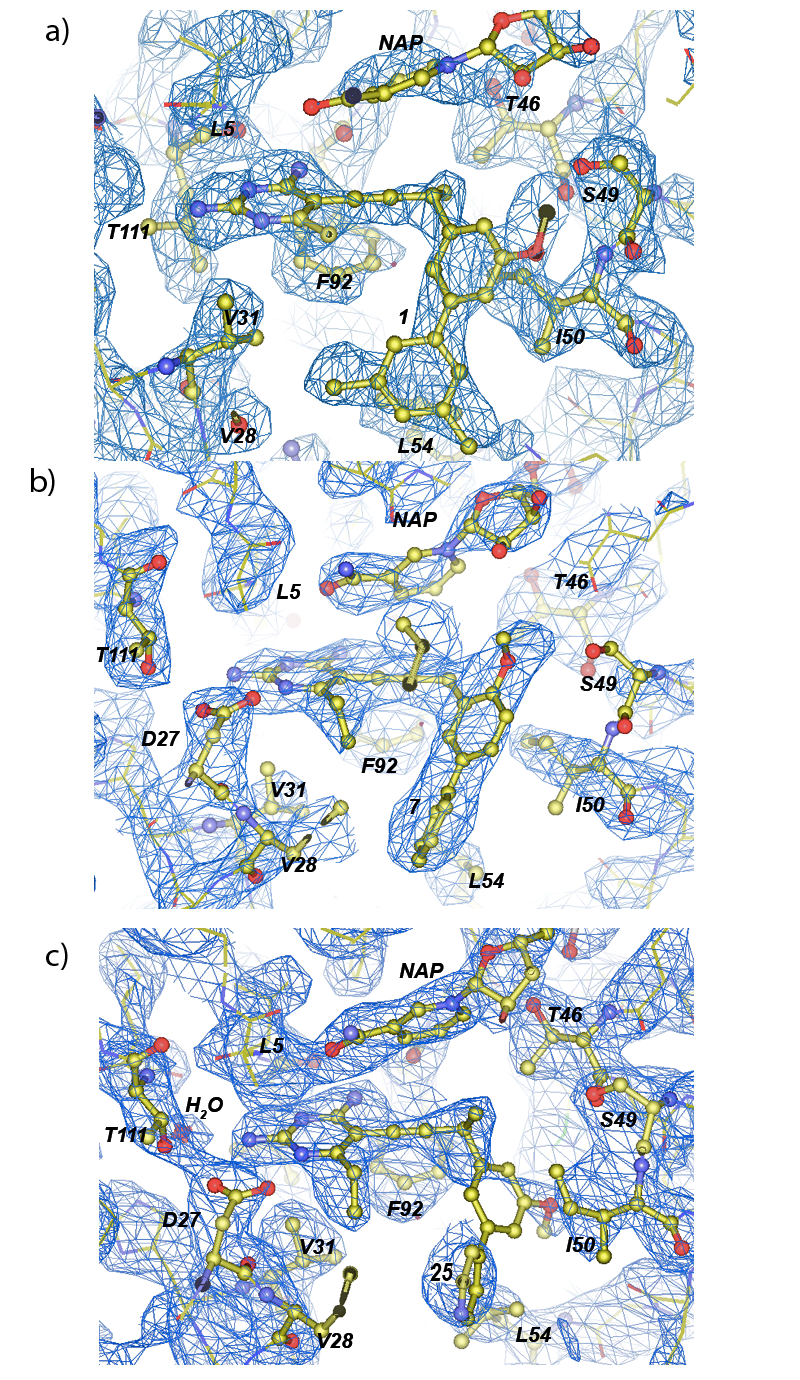

Supplement: Figure S1 — Electron density for the active site and ligands, calculated as Fo-Fc omit maps show a) compound 1 (1.3 σ), b) compound 7 (1.3 σ) and c) compound 25 (1.0 σ). (TIF) [file pone.0029434.s002.tif]
