# Supplementary material for: Toward New Therapeutics for Skin and Soft Tissue Infections: Propargyl-Linked Antifolates Are Potent Inhibitors of MRSA and Streptococcus pyogenes
Source: PLoS One. 2012 Feb 7;7(2):e29434. doi: 10.1371/journal.pone.0029434 (PMC3274548; doi:10.1371/journal.pone.0029434)
Supplement: Table S1 — Statistics for data collection and refinement of crystal structures. (DOC) [file pone.0029434.s003.doc]

Table S1. Statistics for data collection and refinement of crystal structures

| Complex | *Sa: NAP:* ***1*** | *Sa: NAP****: 7*** | *Sa: NAP****: 25*** |
| --- | --- | --- | --- |
| PDB ID | 3F0S | 3SH2 | 3SGY |
| Space group | P6_1_ 22 | P6_1_ | P6_1_ |
| No. Molecules in the asymmetric unit | 2 | 1 | 2 |
| Unit cell (a,b,c in Å) | a=b=78.96,  c=107.42 | a=b=84.62,  c= 103.18 | a=b=78.28,  c=106.92 |
| Resolution (Å) | 37.10 - 2.70 | 37.32 - 3.04 | 42.18 - 2.60 |
| Last shell (Å) | 2.80–2.70 | 3.10–3.04 | 2.64–2.60 |
| *R_sym_*, (last shell) | 0.076 (0.261) | 0.173 (0.490) | 0.082 (0.291) |
| Completeness, % (last shell %) | 92.0 (87.9) | 99.8 (100) | 97.0 (98.8) |
| Unique Reflections | 5169 | 8349 | 10835 |
| Redundancy (last shell) | 5.0 (9.8) | 5.6 (5.4) | 5.7 (5.7) |
| *I/σ* (last shell) | 3.1 (4.7) | 20.2 (3.2) | 31.1 (5.5) |
| **Refinement Statistics** |  |  |  |
| *R*_factor_, *R_free_* | 0.204, 0.269 | 0.218, 0.250 | 0.217, 0.267 |
| Rms deviation bond lengths (Å), angles (°) | 0.012, 1.675 | 0.006, 0.983 | 0.008, 1.246 |
| Average B factors (Å^2^): overall, compound, solvent | 21.09, 24.85, N/A | 57.84, 53.71, N/A | 51.704, 58.45, 43.45 |
| Residues in most favored regions; allowed regions of Ramachandran plot | 88.2, 11.8 | 87.5, 12.5 | 89.7, 10.3 |
